# Over- and under-supply of inpatient rehabilitation after stroke without a post-acute rehabilitation system: a nationwide retrospective cohort study

**DOI:** 10.3389/fneur.2023.1135568

**Published:** 2023-06-16

**Authors:** Suk Won Bae, Junhyun Kwon, Hyung-Ik Shin

**Affiliations:** ^1^Department of Rehabilitation Medicine, Seoul National University Hospital, Seoul, Republic of Korea; ^2^Institute of Health Services Research, Yonsei University College of Medicine, Seoul, Republic of Korea; ^3^Department of Rehabilitation Medicine, Seoul National University College of Medicine, Seoul, Republic of Korea

**Keywords:** stroke, rehabilitation, post-acute phase, length of hospital stay, hospitalization

## Abstract

**Introduction:**

This study aimed to investigate the utilization of post-ischemic stroke rehabilitation prior to the introduction of the post-acute rehabilitation system in South Korea in 2017.

**Methods:**

Medical resources utilized for patients with cerebral infarction hospitalized at Regional Cardio-Cerebrovascular Centers (RCCVCs) of 11 tertiary hospitals were tracked until 2019. Stroke severity was classified according to the National Institutes of Health Stroke Scale (NIHSS), and multivariate regression analysis was performed to analyze factors influencing the length of hospital stay (LOS).

**Results:**

This study included 3,520 patients. Among 939 patients with stroke with moderate or greater severity, 209 (22.3%) returned home after RCCVC discharge without inpatient rehabilitation. Furthermore, 1,455 (56.4%) out of 2,581 patients with minor strokes with NIHSS scores ≤4 were readmitted to another hospital for rehabilitation. The median LOS of patients who received inpatient rehabilitation after RCCVC discharge was 47 days. During the inpatient rehabilitation period, the patients were admitted to 2.7 hospitals on average. The LOS was longer in the lowest-income group, high-severity group, and women.

**Conclusion:**

Before the introduction of the post-acute rehabilitation system, treatment after stroke was both over- and under-supplied, thus delaying home discharge. These results support the development of a post-acute rehabilitation system that defines the patients, duration, and intensity of rehabilitation.

## 1. Introduction

Stroke is known as one of the leading causes of disability worldwide (1–7). Moreover, it poses a great socioeconomic burden to individuals and society (6, 8). Rehabilitation after acute stroke treatment has been demonstrated to reduce the impairment of body function and limitation of activity, thereby facilitating an early return to home (7, 9, 10).

The guidelines for healthcare professionals from the American Heart Association/American Stroke Association emphasize the need for sustained and coordinated efforts for stroke rehabilitation (11). In many countries, specialized rehabilitation units or hospital systems have been developed to provide stroke survivors with rehabilitation treatment in the post-acute period (12, 13). These rehabilitation hospitals dedicated to the post-acute period are often subject to reimbursement tiers, which are based on a case-mix classification determined by disease group, severity, age, comorbidities, and other variables. For example, the prospective payment system in the United States utilizes prepayment tiers mainly determined by functional independence measures and age (14, 15). Japan's convalescent rehabilitation ward system sets an upper limit on the length of hospitalization that is dependent on disease groups. Additionally, post-acute rehabilitation facilities are evaluated by government or private agencies for indicators such as functional gains and returning home rate (16).

In South Korea, a post-acute rehabilitation hospital system was not introduced until 2019 (17), and reimbursement by the National Health Insurance Service (NHIS) was made in the form of fee-for-service for individual physiotherapy and occupational therapy (18). The payment level made to long-term care hospitals (LTCHs) without deduction did not differ from that of acute care hospitals. However, no criteria were established for the target population for medical rehabilitation or the duration of provision. When patients were hospitalized for rehabilitation treatment, there was also no evaluation system for providers, and payment by the NHIS for rehabilitation treatment was guaranteed for up to 2 years after the stroke (19).

South Korea's post-acute rehabilitation system was launched in 2020 (18). Medical institutions adopting the system allow patients to be admitted within 3 months after stroke and provide inpatient rehabilitation programs for up to 6 months. During this period, providing rehabilitation treatment for up to 4 h daily is possible. The rehabilitation physicians decide the types of treatment to be applied (e.g., gait training and swallowing training). Changes in body function, activity level during hospitalization, and return home rate after discharge are monitored (19).

To evaluate the effects of this system in the future, it is necessary to analyze the use of medical resources related to rehabilitation services to set a baseline before its introduction. No previous studies have described the use of rehabilitation medical resources before the introduction of the post-acute rehabilitation system. Hence, the methodology of this study can serve as a useful reference for countries planning to implement a post-acute rehabilitation system in the future to evaluate the effects after the introduction of this system.

This study aimed to investigate the length of hospital stay (LOS) and related factors for rehabilitation treatment after an ischemic stroke in 2017 before the introduction of the post-acute rehabilitation system.

## 2. Materials and methods

### 2.1. Data source

This study tracked the medical resources utilized by patients with ischemic stroke who were hospitalized and discharged from the Regional Cardio-Cerebrovascular Centers (RCCVCs) of 11 tertiary hospitals in 2017 (20). RCCVCs are publicly designated and operated institutions in South Korea for the acute management of thrombolysis in patients with ischemic stroke and ischemic heart disease. Medical information of patients with stroke hospitalized in 11 RCCVCs was registered in the Regional Stroke Center Registry (RSCR) (21). Data were collected from the time of emergency room admission to discharge from the RCCVCs. The RSCR includes information on neurological status at admission and discharge measured by the National Institutes of Health Stroke Scale (NIHSS), medical or surgical interventions, risk factors, and activity levels at admission and discharge measured using the modified Barthel index and modified Rankin scale. For this study, the RSCR data of the patients who were discharged from the RCCVC in 2017 were linked to the data of the relevant patients in the NHIS-National Health Information Database (NHID), and the use of medical resources was tracked until 2019.

The Korean NHIS is a compulsory social health insurance system implemented for all citizens. The NHIS-NHID includes information on demographic and socioeconomic characteristics, births and deaths, healthcare service utilization, and specifics on medical service providers.

### 2.2. Data linkage

The linkage between the RSCR and NHIS-NHID was implemented by the division of big data management within the NHIS, and the resident registration number of each patient was used as a linkage key. To protect personal information, the NHIS provided connected data to the researchers after deleting the resident registration number. The researchers could access the data only in the secure online environment provided by the NHIS.

This study was approved by the Institutional Review Board of Seoul National University Hospital (IRB No. H-2101-139-1192). The requirement for informed consent was waived because the data in the NHIS were anonymized and de-identified (22).

### 2.3. Study population

**Figure 1:**
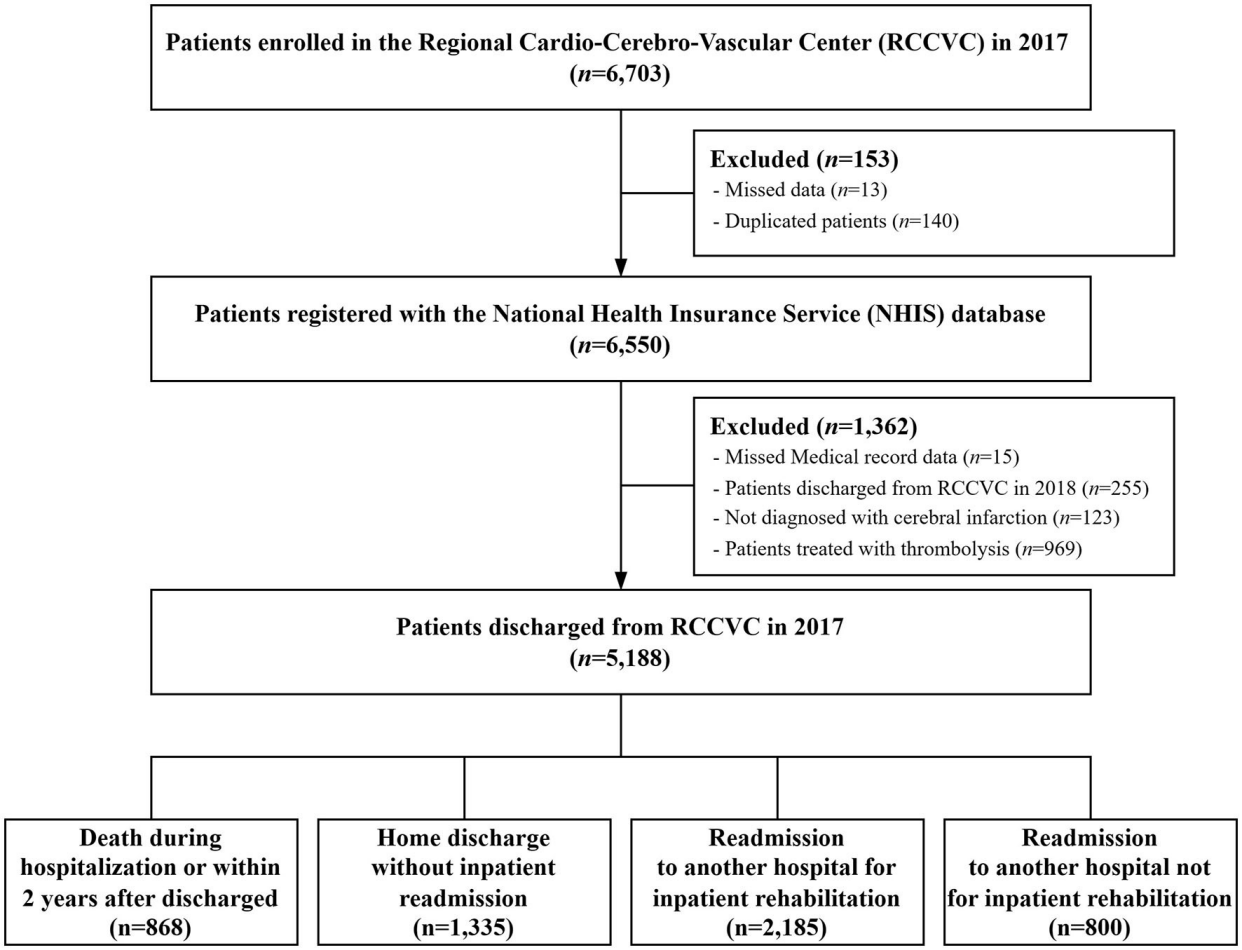
Schematic diagram of the study population.

The study population consisted of 6,703 patients with cerebral infarction (ICD-10 codes I63, I67, I68, and I69) admitted to RCCVCs from 1 January 2017 to 31 December 2017. Among these, 6,550 patients were linked to the NHIS-NHID (linkage rate: 97.7%). To track medical resource utilization during the 2 years after discharge, this study analyzed NHIS-NHID data until 2019. In the 255 patients who were hospitalized in RCCVCs in 2017 but discharged in 2018, a tracking period of 2 years could not be achieved. Therefore, these were excluded from the analysis. In this study, severity was classified according to the initial NIHSS score during hospitalization. Since thrombolysis treatment can affect severity (23), 969 patients who received the intervention were excluded from the analysis. Figure 1 presents a schematic of the study population.

### 2.4. Outcome variables

The primary outcome variable in this study was the LOS from ER admission to home discharge. Only hospitalization for rehabilitation treatment was investigated and presented. When a patient with a stroke was admitted to several hospitals until home discharge, the LOS at each hospital was summed.

Hospital admission after stroke was also assessed. For example, one hospital was utilized when a patient was discharged home from the RCCVC, and two were used when a patient was discharged from an LTCH, followed by home discharge. The total follow-up period lasted until 31 December 2019. Therefore, all patients were followed up for 2 years after discharge from the RCCVCs.

### 2.5. Explanatory variable

The NIHSS was used to assess stroke (24). In this study, the initial NIHSS scores were measured and categorized into four severity groups: minor, 0–4; moderate, 5–15; moderate to severe, 6–20; and severe, 21–42 (5).

Additional explanatory variables included age (<65, 65–74, 75–84, and ≥85 years), sex (male or female), and income level. Income level was classified into four different groups according to the level of NHIS co-payment: quartiles 1–4. As the NHIS-NHID has no separate variable for income, NHIS co-payment was considered the patient's income. The lowest group (1st quartile) corresponded to medical aid, and no health insurance co-payment was recorded in this group (22).

Information on the Charlson Comorbidity Index (CCI) was also collected to evaluate patients' comorbidity status before they were admitted to the RCCVCs. Based on the CCI, the severity of comorbidities was categorized as low (0, 1) or high (≥2) (3).

### 2.6. Statistical analysis

Two groups “not receiving inpatient rehabilitation group (home discharge from RCCVCs)” and “receiving inpatient rehabilitation group (readmission to another hospital for rehabilitation after discharge from RCCVCs)” were compared. We performed a chi-square test and an analysis of variance for the general characteristics of the study population. For the inpatient rehabilitation group, we analyzed the LOS and number of hospital admissions during the 2-year follow-up period after discharge from the RCCVCs. For each variable, the mean and median (interquartile range [IQR]) values were calculated.

Multivariate regression analysis was used to analyze the factors affecting the LOS. Independent variables included the NIHSS score, age, sex, income level, and CCI. All statistical analyses were performed using the SAS statistical software (version 9.4; Statistical Analysis System Institute, Cary, NC, USA).

## 3. Results

Among the 5,188 patients linked to the NHIS-NHID, 868 died during hospitalization or within 2 years after discharge from the RCCVCs. Overall, 800 patients were readmitted to a tertiary hospital for purposes other than inpatient rehabilitation, 315 of whom experienced medical complications (Supplementary Table 1). A total of 240 patients had stroke recurrence, and 245 patients had planned readmissions (Supplementary Table 2). Hence, 1,668 patients were excluded from the analysis.

Analyses were performed for the remaining 3,520 patients (Table 1). Among them, 1,335 patients returned home after discharge from the RCCVCs without inpatient rehabilitation, and 2,185 patients were transferred to another hospital for inpatient rehabilitation. Among the 2,581 patients with minor strokes, 1,455 (56.4%) were readmitted to another hospital for inpatient rehabilitation. Meanwhile, of the 939 patients with moderate severity or above, 209 patients (22.3%) returned home after discharge from the RCCVCs without inpatient rehabilitation.

**Table 1:** General characteristics of the study population.

**Variable**	**Total**		**Not receiving inpatient rehabilitation**		**Receiving inpatient rehabilitation**		P-value^*^
	***N***	**%**	***N***	**%**	***N***	**%**	
Total	3,520	100.0	1,335	100.0	2,185	100.0	
**Median (IQR) length of stay in RCCVC, day**	7	6–12	6	5–9	8	6–14	
Age							<0.001
<65 years	1,281	36.4	577	43.2	704	32.2	
65–74 years	957	27.2	368	27.6	589	27.0	
75–84 years	1,070	30.4	342	25.6	728	33.3	
≥85 years	212	6.0	48	3.6	164	7.5	
Sex							<0.001
Male	2,096	59.5	881	66.0	1,215	55.6	
Female	1,424	40.5	454	34.0	970	44.4	
NIHSS							<0.001
0–4	2,581	73.3	1,126	84.3	1,455	66.6	
5–15	855	24.3	206	15.3	649	29.7	
16–20	60	1.7	2	0.1	58	2.7	
21–42	24	0.7	1	0.1	23	1.1	
Income, KRW							0.107
1st quartile	689	19.6	234	17.5	455	20.8	
2nd quartile	570	16.2	222	16.6	348	15.9	
3rd quartile	780	22.2	315	23.6	465	21.3	
4th quartile	1,421	40.4	538	40.3	883	40.4	
Charlson comorbidity index							<0.001
Low (0–1)	1,996	56.7	863	64.6	1,133	51.9	
High (≥2)	1,524	43.3	472	35.4	1,052	48.1	

IQR, interquartile range; RCCVC, Regional Cardio-Cerebrovascular Center; NIHSS, National Institutes of Health Stroke Scale; KRW, Korean Won.

^*^P-value from χ^2^-test (categorical variables), t-test, or ANOVA (continuous variables).

The median LOS in the RCCVCs was 7 (IQR: 6, 12) days for all 3,520 patients, 6 (IQR: 5, 9) days for the “not receiving inpatient rehabilitation” group, and 8 (IQR: 6, 14) days for the “receiving inpatient rehabilitation” group.

The patients in the inpatient rehabilitation group were older and had a higher severity when measured by the NIHSS than those who did not receive inpatient rehabilitation (*P* < 0.001 and *P* < 0.001, respectively). The proportion of women was higher in the inpatient rehabilitation group (*P* < 0.001).

The median LOS from ER admission to home discharge in the inpatient rehabilitation group was 47 (IQR, 17–196) days. During this period, these patients received rehabilitation treatment while being hospitalized at three hospitals sequentially at a median value (IQR: 2, 4), rather than being admitted to a single hospital (Table 2). Patients with severe stroke had a longer LOS and a higher number of hospital admissions (*P* < 0.001). Female (*P* < 0.001 and *P* = 0.006, respectively) and elderly (*P* < 0.001 and *P* = 0.001, respectively) patients also had a longer LOS and a higher number of hospital admissions. The lowest income level group (Q1) had a longer LOS than that of the other income level groups (*P* = 0.050).

**Table 2:** Length of hospital stay and number of hospital admissions in the inpatient rehabilitation group (*N* = 2,185).

**Variable**	**Length of stay (day)** ^*^				**P-value**	**Number of hospital admissions** ^*^				**P-value**
	**Mean (median)**	**Q1**	**–**	**Q3**		**Mean (median)**	**Q1**	**–**	**Q3**	
Total	191.4 (47)	17	–	196		3.7 (3)	2	–	4	
Age					<0.001					0.001
<65 years	134.8 (30.5)	14	–	113		3.4 (3)	2	–	4	
65–74 years	169.8 (43)	16	–	155		3.8 (3)	2	–	5	
75–84 years	233.0 (62.5)	21	–	302		3.9 (3)	2	–	5	
≥85 years	327.4 (82)	30	–	766.5		4.0 (3)	2	–	4	
Sex					<0.001					0.006
Male	160.1 (36)	15	–	141		3.6 (3)	2	–	4	
Female	230.7 (63)	21	–	279		3.9 (3)	2	–	5	
NIHSS					<0.001					<0.001
0–4	118.0 (29)	14	–	93		3.5 (3)	2	–	4	
5–15	307.9 (128)	39	–	574		4.0 (3)	2	–	5	
16–20	558.8 (719)	117	–	902		4.9 (4)	3	–	6	
21–42	621.0 (845)	224	–	972		4.2 (3)	2	–	5	
Income, KRW					0.050					0.932
1st quartile	226.8 (61)	16	–	276		3.8 (3)	2	–	5	
2nd quartile	191.3 (48)	17	–	223		3.6 (3)	2	–	4	
3rd quartile	176.7 (44)	17	–	171		3.7 (3)	2	–	4	
4th quartile	179.9 (43)	17	–	162		3.7 (3)	2	–	4	
Charlson comorbidity index					0.502					<0.001
Low (0–1)	187.4 (46)	16	–	195		3.5 (3)	2	–	4	
High (≥2)	195.8 (47)	18	–	197.5		3.9 (3)	2	–	5	

Q1, 1st quartile; Q3, 3rd quartile; NIHSS, National Institutes of Health Stroke Scale; KRW, Korean Won.

^*^Including hospitalizations in Regional Cardio-Cerebrovascular Centers.

Table 3 describes the multivariate regression model between LOS and the explanatory variables. In this model, LOS was longer with older age and increasing severity. In addition, the LOS was longer for women and Q1, the lowest income group (*P* = 0.002 and *P* = 0.020, respectively).

**Table 3:** Multivariate regression model for the length of hospital stay in the inpatient rehabilitation group (*N* = 2,185).

**Variable**	**Length of stay (day)** ^*^					
	**Crude model**			**Adjusted model** ^†^		
	β	**SE**	**P-value**	β	**SE**	**P-value**
**Age**						
<65 years	Ref			Ref		
65–74 years	35.0	14.3	0.014	32.0	13.4	0.017
75–84 years	98.2	14.8	<0.001	80.2	14.2	<0.001
≥85 years	192.5	30.9	<0.001	141.1	28.8	<0.001
**Sex**						
Male	Ref			Ref		
Female	70.6	12.8	<0.001	36.0	11.6	0.002
NIHSS						
0–4	Ref			Ref		
5–15	189.9	14.5	<0.001	179.7	14.3	<0.001
16–20	440.8	51.2	<0.001	420.2	51.7	<0.001
21–42	503.0	81.4	<0.001	470.1	83.7	<0.001
**Income, KRW**						
1st quartile	46.9	17.9	0.009	38.4	16.5	0.020
2nd quartile	11.3	18.0	0.529	26.5	17.0	0.120
3rd quartile	−3.2	16.1	0.842	9.7	14.6	0.507
4th quartile	Ref			Ref		
**Charlson comorbidity index**						
Low (0–1)	Ref			Ref		
High (≥2)	8.4	112.5	0.502	1.5	11.7	0.895

SE, standard error; NIHSS, National Institutes of Health Stroke Scale; KRW, Korean Won.

^*^Including hospitalization in Regional Cardio-Cerebrovascular Centers.

^†^Statistically estimated from multivariate regression analyses adjusted for all explanatory variables.

## 4. Discussion

Although there are no standard guidelines on the strategies for rehabilitation treatment according to the severity of the stroke, patients with minor stroke (NIHSS 0–4) may generally undergo outpatient-based rather than intensive inpatient rehabilitation. By contrast, patients with moderate or higher stroke severity often require inpatient rehabilitation during the post-acute period (25). This study investigated the rehabilitation healthcare delivery system before the introduction of the post-acute rehabilitation hospital system and identified that rehabilitation after stroke was not properly performed based on severity (9). Before 2000, Japan had no dedicated system in inpatient facilities to provide early and intensive rehabilitation (26). Prior to 2000, Japan faced the same situation as South Korea.

In the US, inpatient rehabilitation facilities are institutions that provide intensive inpatient rehabilitation treatment after an acute period of 13 diseases, including stroke. Patients can undergo intensive multidisciplinary rehabilitation programs. They receive 3 h of treatment for 5 of 7 consecutive days (18, 27). Patients who have completed functional improvement are subject to an_active return-to-society program, and those whose condition does not improve discontinue rehabilitation treatment and are transferred to an LTCH (18). The Japanese national insurance system introduced kaifukuki (convalescent) rehabilitation wards (KRW) in 2000 (26). Rehabilitation treatment was limited to 3 h per day, and the maximum length of stay for patients with stroke in the KRW was limited to 150 days. The timing of discharge is set when patients reach a plateau in activities of daily living according to an interactive evaluation, which has facilitated the home discharge of patients with stroke with severe disability (16).

In our study, among 939 patients with stroke of moderate severity or above, 209 (22.3%) returned home after discharge from the RCCVCs without inpatient rehabilitation (Table 1). In South Korea, rehabilitation services were reimbursed based on the fee-for-service system even before the post-acute rehabilitation hospital system was introduced. Therefore, rehabilitation treatment may not be implemented in the post-acute period in these groups, not for economic reasons, but because the referral system between medical institutions did not work. Since the median LOS in RCCVCs was 6 days or less and outpatient-based rehabilitation treatment is rarely performed in South Korea, rehabilitation services could be undersupplied for these patients (26).

Meanwhile, among 2,581 patients with minor stroke with an NIHSS score of ≤4, 1,455 (56.4%) were readmitted to another hospital for inpatient rehabilitation after discharge from RCCVCs (Table 1). The average (median) LOS was 118.0 (29) days (Table 2). In this group, rehabilitation treatment could be oversupplied. Therefore, this study shows that rehabilitation services could be over- or under-supplied to patients with stroke without systematic provision of medical rehabilitation in the post-acute period (9).

Another characteristic phenomenon demonstrated in this study was that patients with stroke in the inpatient rehabilitation group visited several hospitals after being discharged from the RCCVCs, resulting in prolonged LOS (9, 28). For example, patients with moderate severity (NIHSS 5–15) who were discharged from RCCVCs and transferred to another hospital to receive rehabilitation services were admitted to two additional hospitals before returning home. The median net LOS was 128 days. This phenomenon may be related to the following issues. First, in South Korea, rehabilitation treatment is mainly provided on an inpatient basis, and outpatient-based rehabilitation treatment is rarely performed after discharge to the home. When receiving rehabilitation treatment on an outpatient basis, the out-of-pocket ratio is higher than that of inpatient rehabilitation. In addition, because of hospitalization fees that can be billed to the NHIS, providers prefer inpatient to outpatient rehabilitation. Regardless of severity, patients with stroke who want rehabilitation treatment after discharge from RCCVCs might have no option other than hospitalization. Second, there were no criteria for determining whether inpatient rehabilitation was required in South Korean NHIS in 2017. Instead, if the hospitalization period exceeds 2 weeks, hospitalization reimbursement is deducted, and after 1 month, it is further deducted (26). However, when patients are admitted to another hospital post-discharge, the deduction rate is not applied cumulatively but rather is reset. Therefore, providers might be concerned with the discharge of patients with stroke but are not interested in whether they are discharged home or admitted to another hospital. Third, among the rehabilitation treatment items based on the fee-for-service reimbursement system, patient/family education and counseling for return to home performed by the rehabilitation team were not included. The absence of these services may have delayed the return to home after the stroke.

Several factors influencing LOS may be related to the specific situation in South Korea rather than being universal. First, the long LOS for women may be because women in South Korea have a higher share of household work and fewer people at home to take care of them (29, 30). Considering these environmental factors, women may be more likely to have difficulty returning home than men after a stroke. Second, the longer LOS in the lowest income group (Q1) can be at least partially explained by the fact that 10–30% of co-payments for medical expenses are not applied to the group (31).

This study has some limitations. First, the NHIS-NHID does not contain information on family members or support. Therefore, information on family support could not be obtained, although this is a known factor that affects LOS (32, 33). Second, the RCCVC system in South Korea performs cardio-cerebrovascular thrombolysis, an emergency procedure. Therefore, patients with other types of stroke, such as cerebral hemorrhage, were excluded from this study. Third, only hospitalization for rehabilitation treatment was investigated and presented, and the rehabilitation treatment itself was not quantitatively analyzed. Because rehabilitation treatment was based on the fee-for-service system as of 2017, it was too complicated to analyze the actual rehabilitation contents and amount of treatment. Fourth, the income level was classified according to the level of NHIS payment. In South Korea, when calculating the NHIS co-payment, the patient's income is determined by salary or tax return, and tax exemption is not included in the calculation. Therefore, patients' incomes in this study may have been underestimated.

In future studies, the use of rehabilitation medical resources and return to home in patients with stroke that occurred after the introduction of the post-acute rehabilitation hospital system in 2020 should be investigated, and the results should be compared with those of this study.

## 5. Conclusion

In conclusion, before the introduction of the post-acute rehabilitation system in South Korea, rehabilitation treatment after stroke was both over- and under-supplied, and return to home was delayed after discharge from acute care hospitals. Returning home after a stroke was the most delayed in the lowest-income group, high-severity group, and female patients. These results support the need for a post-acute rehabilitation system that specifies the subject, duration, and intensity of rehabilitation treatment. The data reported in this study may serve as basic evidence for planning new systems in countries where a post-acute rehabilitation system has not yet been established.

## Data availability statement

The original contributions presented in the study are included in the article/Supplementary material, further inquiries can be directed to the corresponding author.

## Ethics statement

The studies involving human participants were reviewed and approved by the Institutional Review Board of Seoul National University Hospital. Written informed consent from the participants' legal guardian/next of kin was not required to participate in this study in accordance with the national legislation and the institutional requirements.

## Author contributions

SB conceived and planned the study, performed the analysis, wrote the original draft with input from all authors, visualized the results, validated the study, and contributed to the interpretation of the results. JK conceived and planned the study, performed the analysis, wrote the original draft with input from all authors, and contributed to the interpretation of the results. H-IS conceived, planned, validated the study, contributed to the interpretation of the results, and supervised the entire process. All the authors have read and approved the final manuscript.
