# Supplementary material for: Over- and under-supply of inpatient rehabilitation after stroke without a post-acute rehabilitation system: a nationwide retrospective cohort study
Source: Front Neurol. 2023 Jun 16;14:1135568. doi: 10.3389/fneur.2023.1135568 (PMC10313472; doi:10.3389/fneur.2023.1135568)
Supplement: Supplementary file 1 [file Table_1.DOCX]

Supplementary Material

Over- and Undersupply of Inpatient Rehabilitation after Stroke without a Post-Acute Rehabilitation System: A Nationwide Retrospective Cohort Study

Suk Won Bae^1,†^, Junhyun Kwon^1,2,†^, Hyung-Ik Shin^1,3,*^

*** Correspondence:** Hyung-Ik Shin, hyungik1@snu.ac.kr

# Supplementary Tables

**Supplementary Table 1.** List of complications that require admission to a tertiary hospital after discharge from regional cardio-cerebrovascular centers.

| **Classification** | **Diagnosis** | **N*** |
| --- | --- | --- |
| Neurological complication | Transient cerebral ischemic attacks and related syndromes | 39 |
|  | Other headache syndromes; Convulsions, NEC | 8 |
|  | Epilepsy | 7 |
|  | Paraplegia and tetraplegia | 6 |
|  | Facial nerve disorders | 4 |
|  | Other nontraumatic intracranial haemorrhage | 3 |
|  | Parkinson’s disease; Other polyneuropathies | 2 |
|  | Meningitis due to other and unspecified causes; Dystonia; Other extrapyramidal and movement disorders; Migraine | 1 |
| Circulatory complication | Heart failure | 12 |
|  | Angina pectoris; Acute myocardial infarction | 10 |
|  | Occlusion and stenosis of precerebral arteries, not resulting in cerebral infarction | 9 |
|  | Essential (primary) hypertension; Occlusion and stenosis of cerebral arteries, not resulting in cerebral infarction | 7 |
|  | Atherosclerosis | 6 |
|  | Atrial fibrillation and flutter; Intracerebral haemorrhage | 5 |
|  | Chronic ischemic heart disease; Pulmonary embolism | 4 |
|  | Cardiomyopathy; Stroke, not specified as haemorrhage or infarction | 2 |
|  | Rheumatic mitral valve diseases; Atrioventricular and left bundle-branch block; Subarachnoid haemorrhage; Aortic aneurysm and dissection; Other aneurysm and dissection; Hypotension; Complications of cardiac and vascular prosthetic devices, implants and grafts | 1 |
| Respiratory complication | Pneumonia, organism unspecified | 22 |
|  | Bacterial pneumonia, NEC | 9 |
|  | Pneumonitis due to solids and liquids | 6 |
|  | Respiratory tuberculosis, not confirmed bacteriologically or histologically | 5 |
|  | Haemorrhage from respiratory passages | 4 |
|  | Influenza due to identified seasonal influenza virus; Respiratory tuberculosis, bacteriologically  and histologically confirmed | 3 |
|  | Pneumonia due to Streptococcus pneumoniae; Other respiratory disorders | 2 |
|  | Other chronic obstructive pulmonary disease; Asthma; Status asthmaticus; Other interstitial pulmonary diseases; Pleural effusion, NEC; Respiratory failure, NEC | 1 |
| Musculoskeletal complication | Fracture of femur | 13 |
|  | Other necrotizing vasculopathies | 5 |
|  | Seropositive rheumatoid arthritis; Fibroblastic disorders; Fracture of shoulder and upper arm | 3 |
|  | Other rheumatoid arthritis; Gonarthrosis [arthrosis of knee]; Other deforming dorsopathies; Other disorders of muscle; Fracture of rib(s), sternum and thoracic spine; Dislocation, sprain and strain of joints and ligaments of shoulder girdle; Fracture of forearm | 2 |
|  | Gout; Other arthritis; Other inflammatory spondylopathies; Other spondylopathies; Shoulder lesions; Other soft tissue disorders, NEC; Injury of muscle and tendon at shoulder and upper arm level | 1 |
| Nephro-urological complication | Chronic kidney disease | 20 |
|  | Acute tubulo-interstitial nephritis | 6 |
|  | Other disorders of urinary system | 4 |
|  | Other abnormal uterine and vaginal bleeding | 2 |
|  | Unspecified nephritic syndrome; Acute renal failure; Neuromuscular dysfunction of bladder, NEC; Hyperplasia of prostate; Other noninflammatory disorders of uterus, except cervix | 1 |
| Gastrointestinal complication | Other diseases of digestive system | 16 |
|  | Cholelithiasis | 8 |
|  | Duodenal ulcer | 6 |
|  | Cholecystitis; Other gastroenteritis and colitis of infectious and unspecified origin | 5 |
|  | Paralytic ileus and intestinal obstruction without hernia; Diverticular disease of intestine; Other inflammatory liver diseases | 3 |
|  | Gastric ulcer; Gastritis and duodenitis; Acute appendicitis; Other diseases of anus and rectum; Peritonitis; Fibrosis and cirrhosis of liver | 2 |
|  | Other diseases of esophagus; Other functional intestinal disorders; Other diseases of intestine; Alcoholic liver disease; Other diseases of biliary tract; Acute pancreatitis; Postprocedural disorders of digestive system, NEC | 1 |
| Oncological complication | Malignant neoplasm of bronchus and lung | 5 |
|  | Malignant neoplasm of stomach | 4 |
|  | Malignant neoplasm of other and unspecified parts of tongue | 3 |
|  | Malignant neoplasm of nasopharynx; Malignant neoplasm of colon | 2 |
|  | Malignant neoplasm of rectum; Malignant neoplasm of liver and intrahepatic bile ducts; Malignant neoplasm of gallbladder; Malignant neoplasm of larynx; Malignant neoplasm of bladder; Malignant neoplasm of thyroid gland; Secondary malignant neoplasm of respiratory and digestive organs; Other specified types of T/NK-cell lymphoma; Benign neoplasm of colon, rectum, anus and anal canal; Benign neoplasm of meninges; Benign neoplasm of other and unspecified endocrine glands | 1 |
| Others | Disorders of vestibular function; Dizziness and giddiness | 18 |
|  | Type 2 diabetes mellitus | 7 |
|  | Intracranial injury | 6 |
|  | Fever of other and unknown origin; Syncope and collapse | 5 |
|  | Vascular dementia; Congenital malformations of cardiac septa; Fracture of lumbar spine and pelvis | 4 |
|  | Other anemias; Unspecified diabetes mellitus; Abnormalities of gait and mobility; Somnolence, stupor and coma | 3 |
|  | Viral infection of unspecified site; Volume depletion; Other disorders of fluid, electrolyte and acid-base balance; Dementia in Alzheimer’s disease; Unspecified dementia; Delirium, not induced by alcohol and other psychoactive substances; Depressive episode; Vertiginous syndromes in diseases classified elsewhere; Toxic effect of carbon monoxide; Complications of procedures, NEC; Pain in throat and chest; Speech disturbances, NEC; Headache | 2 |
|  | Other sepsis; Streptococcus and staphylococcus as the cause of diseases classified to other chapters; Disorders of lipoprotein metabolism and other lipidemias; Other mental disorders due to brain damage and dysfunction and to physical disease; Mental and behavioral disorders due to use of alcohol; Schizophrenia; Other anxiety disorders; Glaucoma; Paralytic strabismus; Suppurative and unspecified otitis media; Superficial injury of head; Injury of blood vessels at neck level; Open wound of wrist and hand; Medical observation and evaluation for suspected diseases and conditions, ruled out | 1 |

* Including duplicates

**Supplementary Table 2.** Reasons for patients with planned readmissions to tertiary hospitals.

| **Reasons for planned readmission** | **N*** |
| --- | --- |
| Percutaneous transcatheter placement of intracoronary stent – single vessel | 77 |
| Percutaneous intravascular installation of metallic stent – carotid | 43 |
| Percutaneous closure of interatrial septal defect | 36 |
| Percutaneous transluminal angioplasty – others | 29 |
| Percutaneous intravascular installation of metallic stent – others | 17 |
| Percutaneous transcatheter placement of intracoronary stent – additional vessel | 14 |
| PCI for chronic total occlusion | 9 |
| Percutaneous intravascular installation of metallic stent – cerebral | 7 |
| PCI for culprit lesion in acute myocardial infarction | 6 |
| Transluminal atherectomy – carotid artery | 5 |
| Transluminal atherectomy – carotid artery (simple) | 3 |
| Transluminal atherectomy – carotid artery (complex) | 3 |
| Valve replacement – mitral valve | 3 |
| Valve replacement – aortic valve | 3 |
| Reoperation of valvuloplasty – mitral valve | 3 |
| Percutaneous transluminal angioplasty – carotid | 2 |
| Valvuloplasty – mitral valve | 2 |
| Percutaneous transcatheter placement of intracoronary stent | 1 |
| Transcatheter aortic valve implantation – transfemoral, trans-subclavian approach | 1 |
| Percutaneous transluminal angioplasty – cerebral | 1 |
| Operation of atrial septal defect | 1 |
| Open mitral commissurotomy | 1 |
| Valvuloplasty – aortic valve | 1 |
| Sutureless aortic valve replacement | 1 |
| Transluminal atherectomy – others | 1 |

* Including duplicates
